# A Novel Dry Selective Isotropic Atomic Layer Etching of SiGe for Manufacturing Vertical Nanowire Array with Diameter Less than 20 nm

**DOI:** 10.3390/ma13030771

**Published:** 2020-02-07

**Authors:** Junjie Li, Yongliang Li, Na Zhou, Guilei Wang, Qingzhu Zhang, Anyan Du, Yongkui Zhang, Jianfeng Gao, Zhenzhen Kong, Hongxiao Lin, Jinjuan Xiang, Chen Li, Xiaogen Yin, Yangyang Li, Xiaolei Wang, Hong Yang, Xueli Ma, Jianghao Han, Jing Zhang, Tairan Hu, Tao Yang, Junfeng Li, Huaxiang Yin, Huilong Zhu, Wenwu Wang, Henry H. Radamson

**Affiliations:** 1 Key Laboratory of Microelectronics Devices & Integrated Technology, Institute of Microelectronics, Chinese Academy of Sciences, Beijing 100029, China; liyongliang@ime.ac.cn (Y.L.); zhouna@ime.ac.cn (N.Z.); zhangqingzhu@ime.ac.cn (Q.Z.); duanyan@ime.ac.cn (A.D.); zhangyongkui@ime.ac.cn (Y.Z.); gaojianfeng@ime.ac.cn (J.G.); kongzhenzhen@ime.ac.cn (Z.K.); linhongxiao@ime.ac.cn (H.L.); xiangjinjuan@ime.ac.cn (J.X.); lichen2017@ime.ac.cn (C.L.); yinxiaogen@ime.ac.cn (X.Y.); liyangyang@ime.ac.cn (Y.L.); wangxiaolei@ime.ac.cn (X.W.); yanghong@ime.ac.cn (H.Y.); maxueli@ime.ac.cn (X.M.); hanjianghao@ime.ac.cn (J.H.); tyang@ime.ac.cn (T.Y.); lijunfeng@ime.ac.cn (J.L.); yinhuaxiang@ime.ac.cn (H.Y.); zhuhuilong@ime.ac.cn (H.Z.); rad@ime.ac.cn (H.H.R.); 2 Microelectronics Institute, University of Chinese Academy of Sciences, Beijing 100049, China; 3 State Key Laboratory of Advanced Materials for Smart Sensing, General Research Institute for Nonferrous Metals, Beijing 100088, China; 4 College of Electronic and Information Engineering, North China University of Technology, Beijing 100144, China; zhangj@ncut.edu.cn (J.Z.); tairanhu1@gmail.com (T.H.); 5 Department of Electronics Design, Mid Sweden University, Holmgatan 10, 85170 Sundsvall, Sweden

**Keywords:** vertical nanopillar, atomic layer etching, SiGe, field effect transistor, nano device, sensor material

## Abstract

Semiconductor nanowires have great application prospects in field effect transistors and sensors. In this study, the process and challenges of manufacturing vertical SiGe/Si nanowire array by using the conventional lithography and novel dry atomic layer etching technology. The final results demonstrate that vertical nanowires with a diameter less than 20 nm can be obtained. The diameter of nanowires is adjustable with an accuracy error less than 0.3 nm. This technology provides a new way for advanced 3D transistors and sensors.

## 1. Introduction

It has taken few decades to search for materials and device designs for different types of sensors and field-effect transistors. Until now, nanowires (NWs) have offered an excellent platform for gas and bio sensing due to their nano-scale size compatibility to molecule size [1,2]. One issue for this application is how to functionalize the surface of NWs to detect a specific gas molecule with good selectively. In such case, NW transistors could be designed where the carrier transport through the channel is affected and signaled in response to “absorption of” or “interaction with” the gas molecules. For simplicity, these NW sensors are laterally formed on the oxide [3,4,5,6,7]. In these NW transistors, a good Ohmic contacts with low contact resistance, high signal-to-noise, and a good isolation of NW contacts are the main issues.

For many sensor applications, Si-based materials have been widely used but recently different 2D crystals have also been demonstrated. In contrary to Si, SiGe alloys offer a better carrier transport property in presence of compressive strain [8,9,10,11].

For field-effect transistors, in order to reduce the short channel effect (SCE) and power consumption caused by device size shrinkage, lateral and vertical gate-all-around (GAA) field-effect transistors have become a trend to replace FinFet [12,13,14]. Therefore, the manufacturing of precise and controllable size nanowires or nanosheets has become an important technology. In addition, in order to obtain a larger drive current, the channel mobility should be improved. It is also a development trend to use higher mobility SiGe and Ge materials instead of silicon [15,16]. The diameter of nanowires or pillars significantly affects the performance of the device.For sensors, a small size diameter will raise sensitivity because of the higher surface-to-volume ratio [17,18]. For GAA transistors, a small channel diameter will improve gate control and reduce device leakage and power consumption [19,20]. In the conventional bottom-up vertical nanowire preparation method, the diameter of the nanowires is mainly controlled by advanced lithography technology [14,21]. Isotropic selective etching of SiGe is a top-down method to obtain nanowires or nanopillars, and its diameter is controlled by etching and does not depend entirely on photolithography [22]. However, how to accurately control the diameter of nanowires or pillars has become a challenging subject. The commonly used methods for the selective etching of SiGe include wet etching of a mixed solution of oxidant and acid [23,24,25], HCl gaseous reaction [26,27,28], and CF_4_ base gas remote plasma etching [29,30], but the control accuracy is not high enough. The recently reported method of alternating cycles of O_2_ and NF_3_/NH_3_/O_2_, because the self-limitation in each cycle cannot be achieved, and the etching amount per cycle is close to 20 nm, so the etching accuracy is still not high enough [31].

In our previous work, the new CF_4_/O_2_/He reaction system without bias power plasma etching technology was studied, but its etching accuracy still needs to be improved because of its continuous etching characteristics [32]. Then, the method of H_2_O_2_ and BOE alternate cycle self-selective etching of SiGe was studied, and the etching accuracy was remarkably improved. However, the surface tension of the wet solution and the capillary effect determine that its application in 3D high aspect ratio devices will be a big limitation in the future [22].

In this article, a novel SiGe vertical NW in Si/SiGe/ Si structure has been proposed as a sensor structure and the work presents how to apply a dry atomic layer etching (ALE) method to form such structures. The alternating circulation based on O_2_ plasma self-limited oxidation and CF_4_/C_4_F_8_ self-limited selective etching was studied systematically. The profile of nanowires and nanopillars, the relationship between the diameter of nanowires/sheets, and the amount of etching and the number of self-limiting etching cycles were also studied by different types of characterizations. The microstructure after etching was studied by HRSEM (high-resolution scanning electron microscope), HRTEM (High-resolution transmission electron microscope) and EDS (energy dispersive spectrometer) as well as high-resolution x-ray diffraction (HRXRD) [33,34].

## 2. Materials and Methods

**Figure 1:**
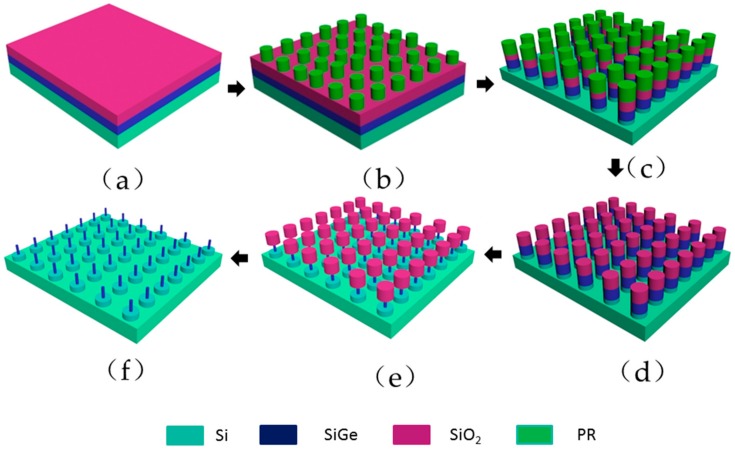
Nano pillar manufacture flow: (**a**) SiGe and SiO_2_ film growth; (**b**) Lithograph obtain PR mask pattern array; (**c**) PR trimming, SiO_2_ hard mask open etching by RIE ether and SiGe/Si anisotropic etching by ICP etcher; (**d**) PR removed by O_2_ plamsma; (**e**) SiGe vertical nanowires formed by atomic layer etching; (**f**) SiO_2_ selectively removed by DHF.

All the materials were performed on 8-inch (100) silicon wafers. The flow of nanopillar manufacture is shown in Figure 1.

Step 1: The film of 60 nm Si_0.72_Ge_0.28_ was grown by using reduced pressure chemical vapor deposition (RPCVD) technique [35,36,37], and then a 60 nm-thick SiO_2_ is grown on the silicon-germanium by plasma enhanced chemical vapor deposition(PECVD).

Step 2: Photoresist lattices with a diameter of 90 nm and a pitch of 500 nm were fabricated on the wafer surface by 248 nm DUV lithography.

Step 3: An 8-inch CCP etcher was used to trim the PR lattice diameter from 90 nm to 70 nm with the gas of CF_4_/O_2_/Ar and opening the SiO_2_ hard mask with the gas of CF_4_/CHF_3_/Ar. Then, SiGe layer and 40 nm Si substrate were anisotropic etched by 8-inch ICP etcher with HBr/O_2_/He gas.

Step 4: PR was removed by a 8-inch ash chamber and wet clean with 100:1 diluted hydrofluoric acid (DHF).

Step 5: An 8-inch ICP etcher with switch process function was used to atomic precisely and selectively isotropic etch SiGe. Then SiGe vertical nanowires were manufactured. 

Step 6: 10:1 DHF was used to remove the SiO_2_ hard mask, and the nano-pillar array was prepared.

**Figure 2:**
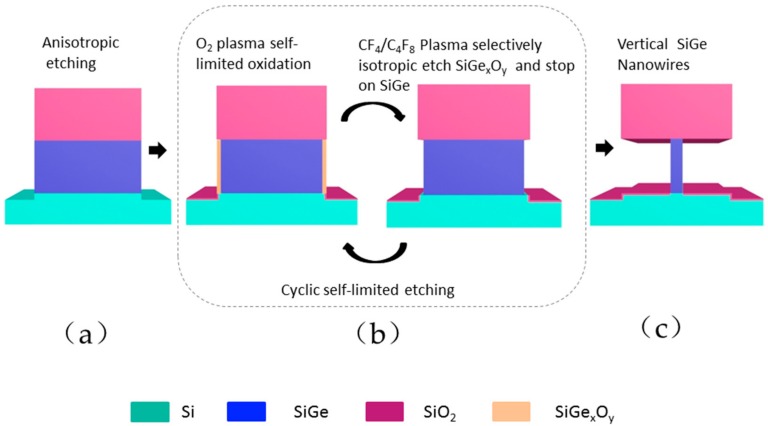
Etching process of vertical nanowires: (**a**) vertical etching with HBr/O_2_/He ICP etcher; (**b**) atomic layer etching: cyclic self-limited oxidation and selective etching of SiGe oxides achieve atomic layer etching accuracy; (**c**) vertical SiGe nanowires with nanometer diameter formed by multiple cycle etching.

The above 5-step etching is a cyclic etching process as show in Figure 2, which is mainly two self-limiting processes: (1) O_2_ plasma self-limiting oxidation: 150 sccm O_2_, 500 W source RF/0 W bias power, treatment can reach oxidation saturation; (2) SiGe_x_O_y_ selective etching: 200 sccm CF_4_/x sccm C_4_F_8_/500 W source RF power/0 W bias power for selective etching of SiGe_x_O_y_ and self-limitation stops on the SiGe surface. Repeat steps (1) and (2) until the diameter of the SiGe nanowire reaches the expected value.

The top-view and cross-section of the sample after etching were analyzed by using high-resolution scanning electron microscope (HRSEM) to check its quality and profile of etching. The defect density in the Si layers after the SiGe release was also analyzed by using high-resolution transmission electron microscope (HRTEM). Energy dispersive spectroscopy (EDS) was employed to determine the element materials of etched layers. The strain relaxation and layer quality were examined by high-resolution x-ray diffraction (HRXRD).

## 3. Results

### 3.1. Study of Atomic Layer Etching SiGe

**Figure 3:**
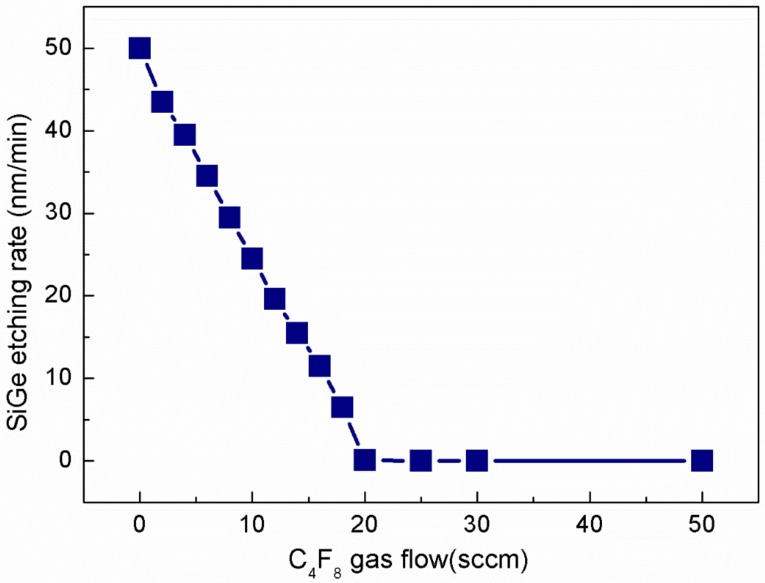
Etching rate of Si_0.72_Ge_0.28_ with different gas flow of C_4_F_8_ and CF_4_ gas flow fixed at 200 sccm.

It is well known that self-limiting sequential reactions are the primary feature of atomic layer etching [38]. According to previous studies, the oxidation of SiGe is self-limiting under certain conditions [39]. Therefore, the focus of this study is how to achieve self-limiting removal of SiGe_x_O_y_ (see Figure 2) and automatically stops on the SiGe layer. We know that SiGe can be oxidized to SiGe_x_O_y_ under O_2_ plasma, and Si can be oxidized to SiO_2_, but before forming these, the oxide needs to open the original covalent bond. The Si–Si bond energy (2.31 eV) > Si–Ge bond energy (2.12 eV), so SiGe alloys are more susceptible to bond oxidation than pure Si [28]. The Si–O bond energy (9.0 eV) is greater than Ge–O bond energy (5.0 eV), the value of the Si–O–Ge bond energy is between the two [40], so using CF-based gas to etch the oxide will form volatile SiF_x_ and GeF_x_ and CO or CO_2_. Hence, increasing the C/F ratio is expected to inhibit the etching on the surface of SiGe, because the absence of O in SiGe cannot make the rich C produce volatile CO or CO_2_. It can be seen from Figure 3 that when the CF_4_ flow rate is fixed at 200 sccm, and the C_4_F_8_ ratio exceeds 20 sccm, SiGe etching has been completely suppressed. Thus, the self-limiting effect of the SiGe_x_O_y_ layer selectively etching is achieved.

**Figure 4:**
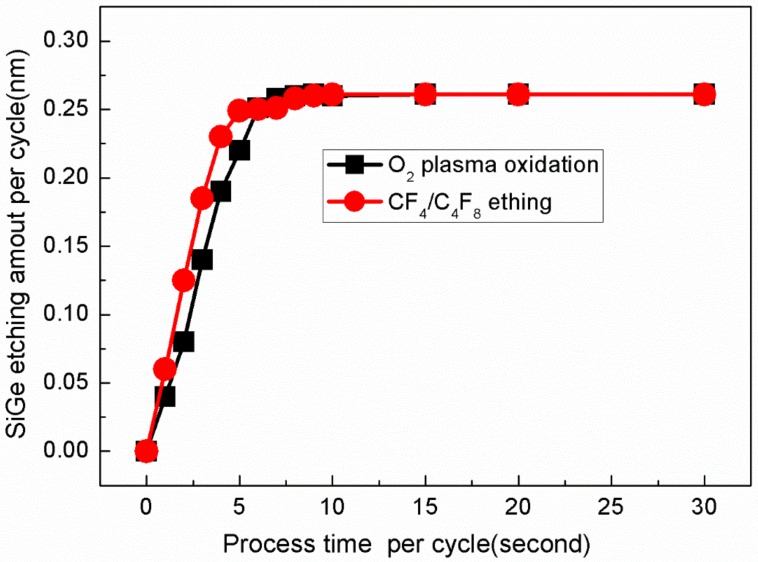
Si_0.72_Ge_0.28_ etching amount of different process time per cycle.

Further experimental data are shown in Figure 4. At 60 mT process pressure and 500 W source RF power, the saturation or self-limiting time of oxidation and selective etching is 6 s and 5 s, respectively.

### 3.2. Study of Nanowire Size and Profile Control

In order to examine the process performance of ALE, the etching conditions of different etching cycles were designed, and then the dimensional and profile characterization of the samples were performed.

**Figure 5:**
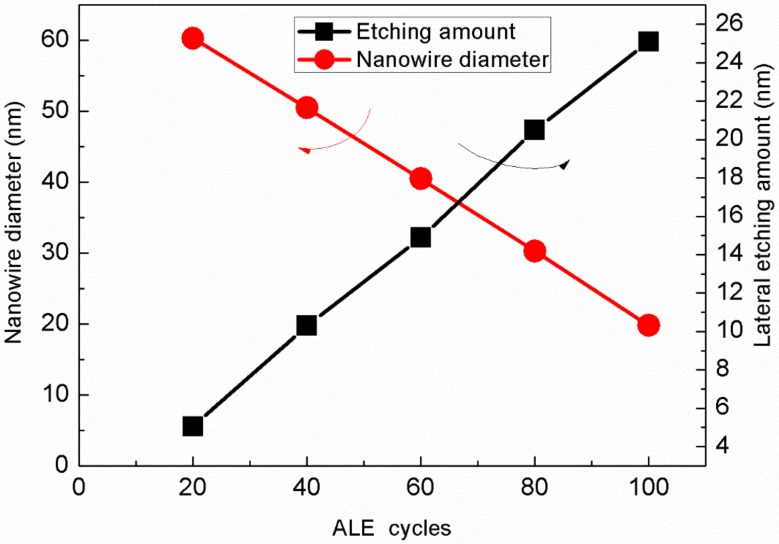
Etching amount and nanowire diameter after different cycles ALE etching.

Firstly, the SiGe nanowire diameter and lateral etching amount of different etching cycles are measured and compared. As shown in Figure 5, the lateral etching amount increases linearly as the number of etching cycles grows, and so do the corresponding SiGe nanometers. The diameter of the wire also decreases linearly as the number of etching cycles reduces. Through 110 cycles of ALE etching, the nanowire diameter can be controlled to less than 20 nm, and the etching amount per cycle is about 0.23 nm, so the control accuracy is very high.

**Figure 6:**
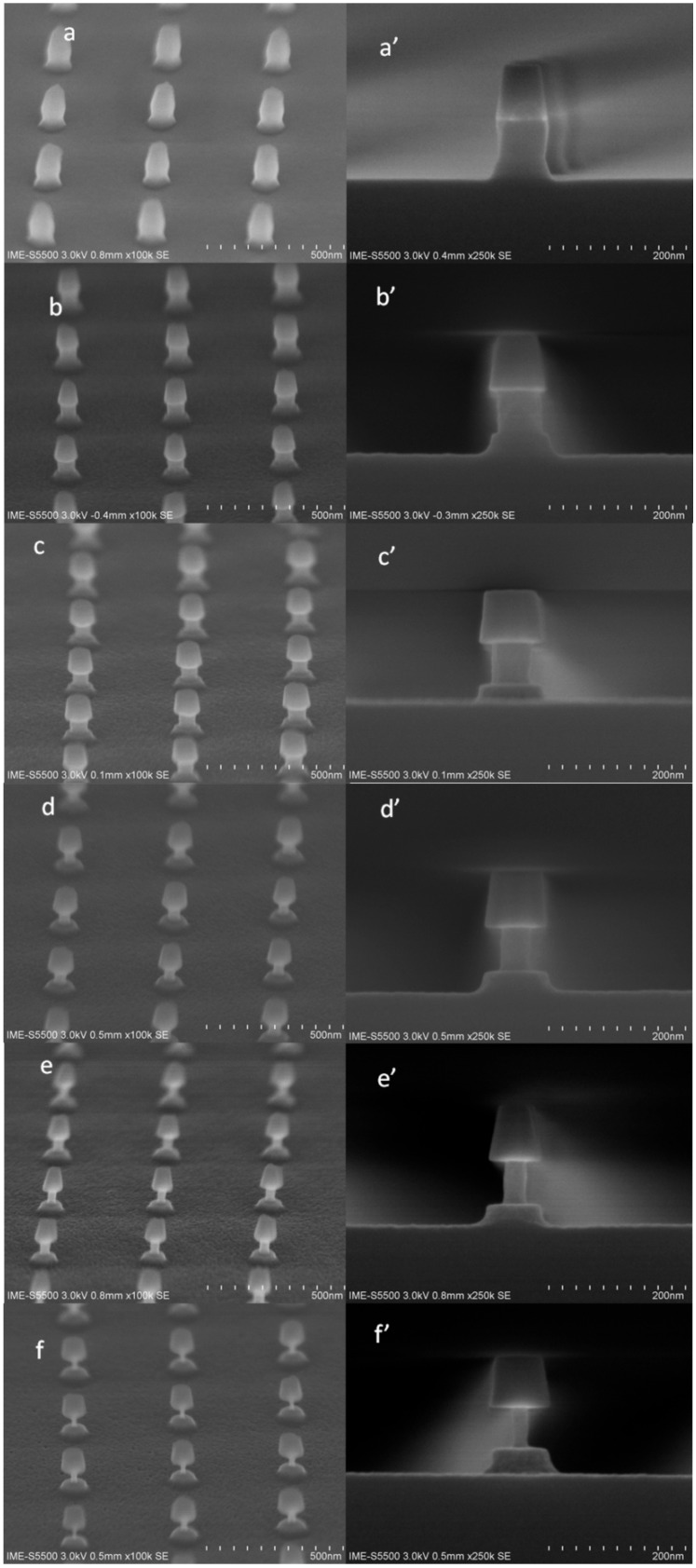
45° top review SEM of profile: (**a**) after anisotropic etching without isotropic ALE etching; (**b**) ALE etching 20 cycles; (**c**) ALE etching 40 cycles; (**d**) ALE etching 60 cycles; (**e**) ALE etching 80 cycles; (**f**) ALE etching 100 cycles; and the cross section SEM images of different samples (**a’**–**f’**), corresponding the sample (**a**–**f**).

Secondly, the morphology of etching was characterized. The SEM results are shown in Figure 6: the dot pattern is relatively uniform (see Figure 6a), the anisotropically etched SiGe part is relatively vertical and the underlying silicon substrate is slightly tilted (see Figure 6a’). This is because the etching of SiGe and Si is a one-step etching. The same etching recipe is used without fine-tuning for different materials. After different cycles of etching, the lattice remains intact (Figure 6b–f), but the diameter of the SiGe part is continuously shrinking, and the shape is still steep and conformal (Figure 6b’–f’)

In general, through ALE etching, the diameter of the nanowires can be flexibly and accurately controlled, and the relatively steep SiGe nanowire morphology can be preserved.

### 3.3. Study Morphology of Nanowire and Nanopillar

**Figure 7:**
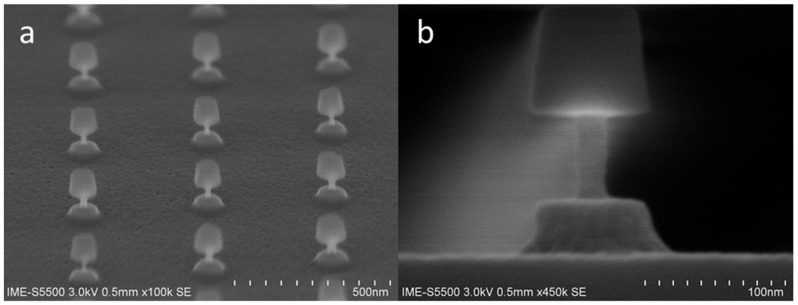
Morphology of nanowires less than 20 nm diameter after 110 cycles etching by ALE: (**a**) 45° top review SEM of profile; (**b**)cross section SEM images.

**Figure 8:**
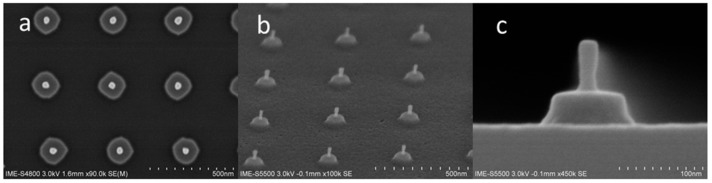
Morphology of nanowires less than 20 nm diameter after 100 cycles ALE and SiO_2_ hard mask remove: (**a**) bird’s-eye top review SEM of profile; (**b**) 45° top review SEM of profile; (**c**) cross section SEM images.

From previous studies, it has been shown that nanowires can be flexibly prepared by using ALE technology without relying on advanced lithography technology. Next, the morphology of nanowires with a diameter of less than 20 nm is mainly studied in terms of SiO_2_ hard mask retention and removal. The result of maintaining the SiO_2_ hard mask is shown in Figure 7. The top view shows that all the lattices in the field of view are intact, and the multiple nanowires have good consistency at low magnifications, and the nanowires have good morphology at high magnifications. 10:1 DHF was used to remove the SiO_2_ hard mask to obtain an independent nano-pillar. The morphological results are shown in Figure 8, which shows that the nano-pillar cross sections are uniform.

### 3.4. Material Quality and Interface Analysis

The layer quality and more detailed information about the parts of the hard mask/SiGe/Si materials have been performed by using HRTEM.

**Figure 9:**
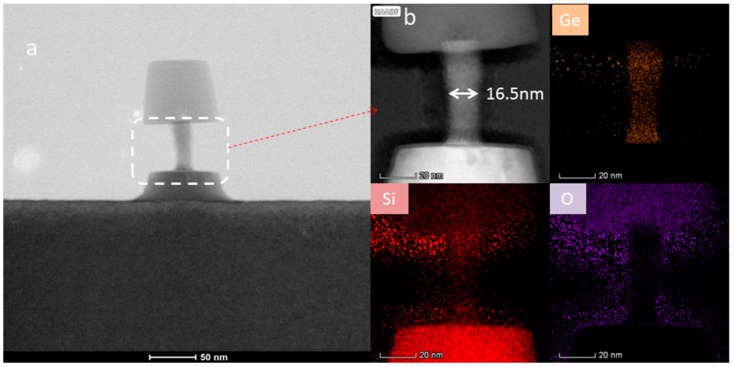
TEM and EDX mapping: (**a**) TEM picture of all structure; (**b**) HRTEM and EDS mapping of near isotropic etching region.

Figure 9 displays the cross-section TEM micrograph of sample in Figure 7b. It is also shown that the nanowires are slightly distorted due to the TEM sample preparation. Figure 9b HRTEM results show that SiGe remains basically perfect in crystal structure and diameter about 16.5 nm. EDS mapping shows that Ge element is mainly in vertical nanowires, Si element is distributed in SiO_2_, SiGe, and Si substrates, O is mainly extant in SiO_2_ hard mask and loading filling material, and the interface of SiGe is also very thin.

**Figure 10:**
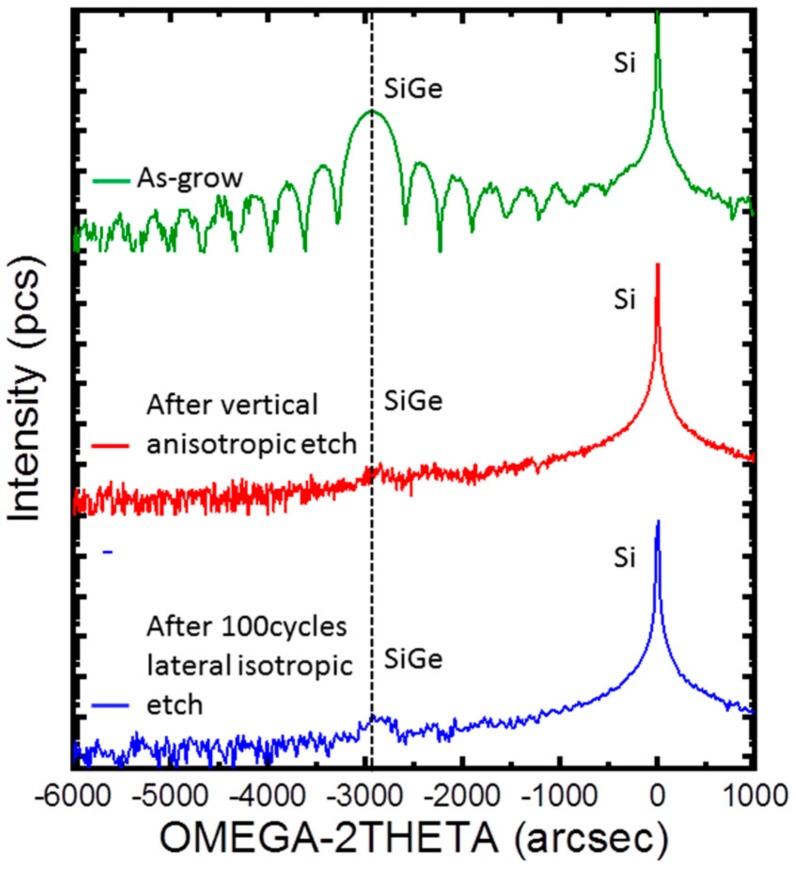
Rocking curves of sample at different steps of processing.

Finally, the integrity of strain in SiGe during etch process has also been studied. It is expected that the strain has impact on the device performance, therefore, strain relaxation has to be studied. In order to obtain a more accurate measurement of strain, reciprocal lattice maps around (113) reflection were performed. Unfortunately, no SiGe peak could be observed due to small size of SiGe layer after etching. Therefore, rocking curves (RCs) measurements were carried out where the x-ray beam has high intensity. Figure 10 shows these RCs measurements. The intensity of SiGe after etching has been remarkably reduced due to removal of large volume of the layer. In these curves, the position of the SiGe has not varied showing the initial strain has been preserved.

The processed vertical SiGe NWs can be later functionalized by e.g., metal oxide to form gate all around (GAA) transistor for future gas sensor.

## 4. Conclusions

With ALE etching, an innovative method for manufacturing nanowires was obtained. The results show that the diameter of the nanowires can be flexibly adjusted from 60 nm to 16.5 nm, and the morphology is under precise control, the microstructure is complete, and there is also no obvious etching residue contamination on the interface. The ALE method could be co-used with advanced lithography (such as initial line width control to <45 nm), and it will be expected to obtain much smaller nanowires with high density in the future. This technique will have very wide and promising applications in sensors.
